# Maternal and Neonatal Infection With *Salmonella heidelberg* : A Case Report

**DOI:** 10.1155/S1064744993000110

**Published:** 1993

**Authors:** Kevin A. Ault, Maureen Kennedy, Muhieddine A.-F. Seoud, Rosemary Reiss

**Affiliations:** 1 Department of Gynecology and Obstetrics University of Kansas Medical Center Kansas City KS USA; 2 Department of Obstetrics and Gynecology Ohio State University Columbus OH USA; 3 Department of Gynecology and Obstetrics University of Kansas School of Medicine, 3901 Rainbow Blvd. Kansas City KS 66160-7316 USA

## Abstract

Maternal and neonatal infections with *Salmonella typhi* have been well documented. There are only two previous case reports of intrauterine infection with non-typhoidal species. This paper presents a third case of maternal septicemia followed by neonatal sepsis with *Salmonella heidelberg*.


Infectious Diseases in Obstetrics and Gynecology 1:46–48 (1993)
© 1993 Wiley-Liss, Inc.

### Maternal and Neonatal Infection With *Salmonella heidelberg*: A Case Report

**Kevin A. Ault, Maureen Kennedy, Muhieddine A.-F. Seoud, and Rosemary Reiss**

*Department of Gynecology and Obstetrics, University of Kansas Medical Center, Kansas City, KS (K.A.A., M.A.-F.S.) and Department of Obstetrics and Gynecology, Ohio State University, Columbus, OH (M.K., R.R.)*

ABSTRACT

**Maternal and neonatal infections with *Salmonella typhi* have been well documented. There are only two previous case reports of intrauterine infection with non-typhoidal species. This paper presents a third case of maternal septicemia followed by neonatal sepsis with *Salmonella heidelberg*.**
© 1993 Wiley-Liss, Inc.

KEY WORDS

Amnionitis, salmonella, premature rupture of membranes

*Salmonella* is a gram-negative bacillus of the family Enterobacteriaceae. Salmonella species are ubiquitous common pathogens in both humans and animals. Types of infection include asymptomatic carriage, gastroenteritis, enteric fever, meningitis, osteomyelitis, and septicemia. Transmission is fecal-oral, and the peak incidence occurs in infants aged 2 through 6 months.[1]

Neonatal infections due to *Salmonella* are well documented.[2] The spread is typically epidemic, with premature infants at higher risk, and the index case is often a mother. Vertical transmission during pregnancy has been documented with identification of *Salmonella typhi* in the amniotic fluid.[3] Case reports of typhoid fever in pregnant women reflect the serious nature and lengthy differential diagnosis of this illness.[4–7]

Serious, non-typhoidal *Salmonella* infections in mothers appear to be less common. Roberts and Wilkins reported the results of screening over 30,000 women for *Salmonella* upon their presentation for delivery.[8] Sixty (0.2%) women were *Salmonella* excreters; the majority were asymptomatic. Two cases of intrauterine infection with presumed transplacental spread have been reported.[9,10] We report here an unusual case of maternal bacteremia with subsequent neonatal infection due to *Salmonella heidelberg*.

### CASE REPORT

The patient was a 25 year old black woman, gravida four, para two, abortus one, at 28 weeks of gestation. Her chief complaint was leakage of fluid per vagina for 2 days with fever and chills. Her antenatal course was otherwise unremarkable. Her rectal temperature on admission was 39°C, and her pulse was 124. She denied any cough, abdominal pain, diarrhea, or neck stiffness. No localizing signs of infection were found on physical examination, and the uterus was noted to be non-tender. Sterile speculum examination confirmed the rupture of membranes, and her cervix appeared closed. The initial leukocyte count was 11,800/$mm^3$, with 85% segmented neutrophils and 5%

**Address correspondence/reprint requests to Dr. Kevin A. Ault, Department of Gynecology and Obstetrics, University of Kansas School of Medicine, 3901 Rainbow Blvd., Kansas City, KS 66160-7316.**

Received October 1, 1992

Accepted November 17, 1992

bands; the hemoglobin was 10.9 g/dl. One hour later, her rectal temperature was 43°C, and she continued to have chills. She was presumed to have prolonged rupture of membranes with choriamnionitis. After obtaining urinary, cervical, and blood cultures, she was started on gentamicin and clindamycin intravenously, as well as acetaminophen and a cooling blanket; labor was induced with dilute oxytocin. Ampicillin was not given secondary to a penicillin allergy.

Labor progressed rapidly, and 6 hours later she delivered vaginally a live male neonate weighing 1,240 g. Apgar scores were 5 and 5 at 1 and 5 minutes, respectively, and an umbilical vein pH was 7.33. The mother was noted to be afebrile at the time of delivery. The preliminary report on the blood cultures was *Salmonella*. The human immunodeficiency virus (HIV) test was negative, and hemoglobin electrophoresis was consistent with sickle cell trait. At the advice of an infectious disease consultant, the antibiotic was changed to oral trimethoprim-sulfamethoxazole. The patient developed diarrhea on postpartum day 2. Cultures of the placenta, maternal blood, and stool were positive for *Salmonella,* serogroup B. She remained afebrile and was discharged on the fourth postpartum day.

The newborn was admitted to the neonatal intensive care unit, intubated, placed on a ventilator, and given gentamicin and ampicillin. He was given artificial surfactant in the first few hours of life. Initial chest x-ray showed opacification bilaterally. Initial blood and respiratory cultures were negative, and the newborn was weaned from the ventilator slowly over the first week of life. Intravenous antibiotics were given for 10 days and then stopped. The newborn was extubated on day 10. Twelve hours after stopping the antibiotics, he developed bradycardia, apnea, and hypoxemia, requiring ventilatory assistance. Leukocytosis was noted, and blood cultures, along with lumbar puncture, were performed. Chest x-ray showed no consolidations. Intravenous ampicillin and gentamicin were started. Twenty-four hours after blood cultures were done, gram-negative rods were detected. Eventually, blood, cerebrospinal fluid, and stool all grew *Salmonella,* serogroup B. The antibiotics were changed to intravenous ceftazidime with ampicillin. The infant gradually improved on this regimen and was soon re-extubated. He eventually developed hydrocephaly, which required a shunt, and bilateral hydroceles requiring herniorrhaphy and orchidopexy. The child was released from the hospital after approximately 3 months. All follow-up cultures were negative for *Salmonella.*

Samples of the bacteria from the maternal and neonatal blood cultures were sent to the local public health department and the Centers for Disease Control and Prevention in Atlanta, GA. The serotype was identified as *S. heidelberg.* In retrospect, the mother reported eating partially cooked chicken several days prior to admission, and this chicken had been purchased directly from a local farm.

### DISCUSSION

*Salmonella typhi* has been shown to cause serious maternal infection, with transplacental spread to the fetus. Systemic disease in adults is often due to *S. typhi* and *paratyphi,* whereas childhood disease is due to a wider variety of serotypes.[2] Most of these childhood infections are cases of bacteremia and gastroenteritis in the first year of life.[1]

We have previously reported on maternal and neonatal infection with *S. typhi.*[7] Both vertical and horizontal transmission have been hypothesized, and positive amniotic and cervical cultures have been reported.[3] The placenta in this case did not show inflammation of membranes; however, it showed suppurative villitis. Scialli et al.[10] presented a case of second-trimester fetal loss associated with group C1 *Salmonella.* Dalaker et al.[9] also reported a case of septic abortion due to *S. enteritidis.* It is likely our case represents maternal sepsis from an enteric source with transplacental spread to the fetus. The neonatal infection on day 10 of life was felt to be secondary to an incompletely treated neonatal septicemia acquired in utero. The infant was in strict enteric isolation throughout the first several weeks of life. There were no other temporally related cases of *Salmonella* in the intensive care unit. Scialli et al.[10] recommend that pregnant women with diarrhea illness be screened for *Salmonella.* We agree with this conclusion, but we realize the difficulty in finding an etiological agent for infectious diarrhea. However, in the setting of maternal sepsis with or without gastrointestinal complaints, it is prudent to consider all *Salmonella* species in the differential diagnosis. Identifying a history of ingestion of contaminated food or possibly a history of immunodeficiency or hemoglobinopathy may help identify mothers at risk for *Salmonella* sepsis and lead to prompt and appropriate therapy.

### REFERENCES

1. Hyams JS, Durbin WA, Grand RI, Goldman DA: *Salmonella* bacteremia in the first year of life. Pediatrics 96:57–59, 1980.
2. Remington DS, Klein S: Microorganisms responsible for neonatal diarrhea. In: Infectious Diseases of the Fetus and Newborn Infant. WB Saunders Company, Philadelphia, PA. 1990, third edition: 920–6.
3. Awadalla SG, Mercer LJ, Brown LG: Pregnancy complicated by intra-amnionitic infection by *Salmonella typhi*. Obstet Gynecol 65:305–315, 1985.
4. Amster R, Lessing JB, Daffa AJ, Peyser MR: Case report: Typhoid fever complicating pregnancy. Acta Obstet Gynecol Scand 64:685–686, 1985.
5. Dildy GA, Martins MG, Faro S, Lee W: Typhoid fever in pregnancy; a case report. J Reprod Med 35:273–276, 1990.
6. Duff P, Engelsgjerd B; Typhoid fever on an obstetrics and gynecology service. Am J Obstet Gynecol 145:113–114, 1983.
7. Seoud M, Saade G, Uwaydah M, Azoury R: Typhoid fever in pregnancy. Obstet Gynecol 71:711–714, 1988.
8. Roberts C, Wilkins EGL: *Salmonella* screening of pregnant women. J Hosp Infect 10:67–72, 1987.
9. Dalaker K, Andersen M, Louslett K, Reuhaug A, Berdal B: Case report—septic abortion caused by *Salmonella enteritidis*. Acta Obstet Gynecol Scand 67:185–186, 1988.
10. Scialli A, Rarick T: Salmonella sepsis and second trimester pregnancy loss. Obstet Gynecol 79:820–821, 1992.

## References

[PDF_00187] Amster R., Lessing J. B., Jaffa A. J., Peyser M. R. (1985). Typhoid fever complicating pregnancy.. Acta Obstet Gynecol Scand.

[PDF_00200] Dalaker K., Andersen B. M., Løvslett K., Revhaug A., Berdal B. (1988). Septic abortion caused by Salmonella enteritidis.. Acta Obstet Gynecol Scand.

[PDF_00190] Dildy G. A., Martens M. G., Faro S., Lee W. (1990). Typhoid fever in pregnancy. A case report.. J Reprod Med.

[PDF_00193] Duff P., Engelsgjerd B. (1983). Typhoid fever on an obstetrics-gynecology service.. Am J Obstet Gynecol.

[PDF_00177] Hyams J. S., Durbin W. A., Grand R. J., Goldmann D. A. (1980). Salmonella bacteremia in the first year of life.. J Pediatr.

[PDF_00198] Roberts C., Wilkins E. G. (1987). Salmonella screening of pregnant women.. J Hosp Infect.

[PDF_00203] Scialli A. R., Rarick T. L. (1992). Salmonella sepsis and second-trimester pregnancy loss.. Obstet Gynecol.

[PDF_00196] Seoud M., Saade G., Uwaydah M., Azoury R. (1988). Typhoid fever in pregnancy.. Obstet Gynecol.

